# Identification and Characterization of the Glucose-6-Phosphate Dehydrogenase Gene Family in the Para Rubber Tree, *Hevea brasiliensis*

**DOI:** 10.3389/fpls.2016.00215

**Published:** 2016-02-25

**Authors:** Xiangyu Long, Bin He, Yongjun Fang, Chaorong Tang

**Affiliations:** Rubber Research Institute, Chinese Academy of Tropical Agricultural SciencesDanzhou, China

**Keywords:** glucose-6-phosphate dehydrogenase, expression, latex regeneration, abiotic stresses, *Hevea brasiliensis*

## Abstract

As a key enzyme in the pentose phosphate pathway (PPP), glucose-6-phosphate dehydrogenase (G6PDH) provides nicotinamide adenine dinucleotide phosphate (NADPH) and intermediary metabolites for rubber biosynthesis, and plays an important role in plant development and stress responses. In this study, four *Hevea brasiliensis* (Para rubber tree) *G6PDH* genes (*HbG6PDH1 to 4*) were identified and cloned using a genome-wide scanning approach. All four *HbG6PDH* genes encode functional G6PDH enzymes as shown by heterologous expression in *E. coli*. Phylogeny analysis and subcellular localization prediction show that *HbG6PDH3* is a cytosolic isoform, while the other three genes (*HbG6PDH1, 2* and *4*) are plastidic isoforms. The subcellular locations of *HbG6PDH3* and *4*, two latex-abundant isoforms were further verified by transient expression in rice protoplasts. Enzyme activity assay and expression analysis showed HbG6PDH3 and 4 were implicated in PPP during latex regeneration, and to influence rubber production positively in rubber tree. The cytosolic HbG6PDH3 is a predominant isoform in latex, implying a principal role for this isoform in controlling carbon flow and NADPH production in the PPP during latex regeneration. The expression pattern of plastidic *HbG6PDH4* correlates well with the degree of tapping panel dryness, a physiological disorder that stops the flow of latex from affected rubber trees. In addition, the four *HbG6PDHs* responded to temperature and drought stresses in root, bark, and leaves, implicating their roles in maintaining redox balance and defending against oxidative stress.

## Introduction

In plants, the pentose phosphate pathway (PPP) is a principal glycometabolism pathway that plays an important role in growth, development, and physiological stresses. The PPP produces nicotinamide adenine dinucleotide phosphate (NADPH) for reductive biosyntheses of metabolic products including fatty acids and amino acids, as well as many intermediary metabolites such as nucleotides (von Schaewen et al., 1995; Dennis et al., 1997; Wakao and Benning, 2005; Wakao et al., 2008). Glucose-6-phosphate dehydrogenase (G6PDH) is recognized as a key enzyme of the plant PPP pathway, and is involved in regulating the metabolic rates of many physiological processes (Kruger and von Schaewen, 2003). According to their subcellular locations, G6PDH molecular variants are divided into cytosolic and plastidic isoforms, both of which are encoded by nuclear genes (Schnarrenberger et al., 1973; von Schaewen et al., 1995; Kruger and von Schaewen, 2003). The *G6PDH* genes have been identified in several plants including potato (Graeve et al., 1994; von Schaewen et al., 1995; Wendt et al., 2000), *Populus* (Lin et al., 2005, 2013), wheat (Nemoto and Sasakuma, 2000), rice (Zhang et al., 2013), barley (Cardi et al., 2013, 2015), soybean (Liu et al., 2013), and *Arabidopsis* (Wakao and Benning, 2005; Wakao et al., 2008; Siddappaji et al., 2013), and their involvement in growth and development events including seed germination (Pu et al., 1994; Han et al., 1998) and oil accumulation (Wakao et al., 2008) has been reported. *G6PDH* genes also respond to various environmental stresses including salt (Nemoto and Sasakuma, 2000; Wang et al., 2008; Zhang et al., 2013; Cardi et al., 2015), drought (Scharte et al., 2009; Liu et al., 2013), heavy metals (Ślaski et al., 1996), and low temperature (Lin et al., 2013; Yang et al., 2014). For years, the studies of G6PDHs have been mainly focused on aspects of transcription and activity analysis under various stresses, stressing their roles in maintaining cell redox balance to enhance stress resistance in plants.

The Para rubber tree, *Hevea brasiliensis*, yields natural rubber (*cis*-1, 4-polyisoprene), which is an important industrial and strategic raw material. Latex (the cytoplasm of rubber-producing laticifers), flowing from severed laticifers in the bark of rubber tree after tapping (the method of rubber harvesting), is the harvested product (Metcalfe, 1967; Lewinsohn, 1991). During every tapping commonly with 2- or 3-day intervals, rubber tree discharges and loses a large amount of latex (more than 100 ml in high yielding cultivars), about one third of which is natural rubber. This lost rubber and other latex constituents including sugars, nucleic acids and proteins are regenerated within the laticifers by the time the tree is next tapped. In laticifers, glycometabolism supplies carbon and energy for rubber regeneration, and its efficiency affects yield production in rubber tree (Tupy, 1973; Jacob et al., 1988; Bouteau et al., 1991, 1992; Silpi et al., 2006, 2007). During latex regeneration, the PPP supplies NADPH for the reducing reactions in rubber biosynthesis, and supplies pentose for nucleic acid synthesis (Lynen, 1969). Various environmental factors such as cold, wind, disease, and especially wounding stresses from tapping affect rubber yield (Sookmark et al., 2002; Venkatachalam et al., 2009; Li et al., 2010; Gebelin et al., 2013; Long et al., 2015). The act of tapping the tree inevitably imposes a form of wounding stress that leads to peroxide accumulation in latex. As a reducing agent, NADPH is thought to act in clearing the superfluous peroxide to sustain the redox balance when trees experience physiological stress. As a rate-limiting enzyme, G6PDH plays an important role in maintaining the carbon flow and NADPH production in the PPP. Hitherto, there is little information available regarding the genes encoding G6PDHs in rubber tree. An analysis of the *G6PDH* family, including its expression and enzymatic activity will improve the understanding of the physiological roles of PPP in rubber tree.

In this study, four *G6PDH* genes of *H. brasiliensis* (*HbG6PDH1 to 4*) comprising one cytosolic and three plastidic isoforms were identified and cloned for the first time. Analyses of enzyme activity and expression patterns suggested that these HbG6PDHs are involved in PPP during latex regeneration, of which the cytosolic HbG6PDH3 is the major isoform that plays a role in the PPP of laticifers and in mitigating the effects of oxidative stress.

## Materials and Methods

### Plant Materials

Reyan7-33-97 (CATAS7-33-97 or RY7-33-97) rubber trees (*H. brasiliensis*) selected for this study were cultivated at the experimental plantation of the Rubber Research Institute of Chinese Academy of Tropical Agricultural Sciences (Danzhou, Hainan, China). These trees were tapped every three days for latex collection in a half spiral cut. To study the tissue expression patterns of the *HbG6PDH*s, seven tissues (latex, leaves, bud, seed, male flower, female flower, and bark) were collected for RNA extraction from 10-year-old mature trees of Reyan7-33-97 that had been tapped over the proceeding past 2 years. Trees of the same cultivar were used to examine the effects of various plant hormones on *HbG6PDH*s. To analyze the effect of tapping and wounding on *HbG6PDH*s expression levels, 8-year-old mature virgin trees (untapped trees) were selected.

### Isolation of *HbG6PDH*s Genes

To isolate and identify the *HbG6PDH* sequences, the *G6PDH* genes of *A. thaliana*, *P. trichocarpa*, and *O. sativa* were used as queries to search against the transcriptome database of *H. brasiliensis*. Based on the sequences of resulting contigs, multiple pairs of primers were designed and used to amplify the cDNAs of *HbG6PDH* (**Table 1**). The PCR products were cloned into the pMD18-T cloning vectors (TaKaRa Biotechnology, Dalian, China), and then transformed into *Escherichia coli* DH5α cells. The obtained full-length cDNA sequences were used in BLAST search and other bioinformatic analysis using the NCBI database.

**Table 1:** Primer sequences used in this paper.

Primer code	Primer sequence	Usage
HbG6PDH1-F	5′-AAC TCC TTG TTC TCT TCT TCA CCA ACT TCT C-3′	Full-length cDNA amplification for *HbG6PDHs*
HbG6PDH1-R	5′-ATA AAC TCC TCT ATT CAT AAA TCT CAC CGC TAA GA-3′	
HbG6PDH2-F	5′-TAG CAT TTC TGG GAA TTC TCT TCA CCA TTA CCT-3′	
HbG6PDH2-R	5′-GAA ATA TAC CAT CAA CCA CAA GAG TGT ATT GTA ACT C-3′	
HbG6PDH3-F	5′-ATG CAA TTG AAT TCT CTT TTG CAG AGC TGA G-3′	
HbG6PDH3-R	5′-ATG CTT ATT ATT AGC AAA CTG AAG TCC TTC ATG TTG G-3′	
HbG6PDH4-F	5′-CAA TGC AGT GAA GTG GGA CCC AAA TTC T-3′	
HbG6PDH4-R	5′-GCC AGA ATT GGA GAG ACT ATA TCA TGT TGT TTG C-3′	
HbG6PDH1-R-F	5′-CGG GGT ACC GAT GCC AAG AAA ATA TGG TCT CTC CAC ATG G-3′	Recombinant proteins for HbG6PDHs
HbG6PDH1-R-R	5′-CGC GGA TCC CTA TTC ATA AAT CTC ACC GCT AAG ATC TCC CC-3′	
HbG6PDH2-R-F	5′-CGG GGT ACC GAT GGC GCC CCT TTC TTC AAT ACA TTG T-3′	
HbG6PDH2-R-R	5′-CGC GGA TCC TTA CTG TTC TAT GCC AAG GTC TCC CCA-3′	
HbG6PDH3-R-F	5′-CCC GCG GAT CCT CAA ATA TTT CCA TTC AAC AAG GTA GC-3′	
HbG6PDH3-R-R	5′-CGG GGT ACC GAT GGG TTC GGG TCA GTG GTT AAT-3′	
HbG6PDH4-R-F	5′-CCG GAA TTC CTA CAG TGT GGG AGG AAT CCA GAT ATA GCC A-3′	
HbG6PDH4-R-R	5′-CGG GGT ACC GAT GTC GAT GTC AAT TTC ATA TTT ATC-3′	
HbG6PDH3-L-F	5′-CAG TGG TCT CAC AAC ATG GGT TCG GGT CAG TGG TT-3′	Localization for HbG6PDHs
HbG6PDH3-L-R	5′-CAG TGG TCT CAT ACA CAG TGT GGG AGG AAT CCA GA-3′	
HbG6PDH4-L-F	5′-CAG TGG TCTCAC AAC ATG TCG ATG TCA ATT TCA TA-3′	
HbG6PDH4-L-R	5′-CAG TGG TCT CAT ACA GTC GTC TGC CCA CCG AAC CC-3′	
HbG6PDH1-Q-F	5′-GGG CAC TAT TCA CAC CAT TGT TA-3′	Real-time PCR for *HbG6PDHs*
HbG6PDH1-Q-R	5′-CTA TAC ACC ACA GCT CCA ATA AAC T-3′	
HbG6PDH2-Q-F	5′-CTT GAA GAG AAA AAG ATT ATT CCT GAG TAC-3′	
HbG6PDH2-Q-R	5′-GGT TAA ACA ACA TTC CCA GAT TTA CTG-3′	
HbG6PDH3-Q-F	5′-AGG TTC TTC AAT CAG TAC TTC CAA T-3′	
HbG6PDH3-Q-R	5′-AAC GCC TTC CCA TCT TTC ATT AT-3′	
HbG6PDH4-Q-F	5′-TCA TCA AAC ACA GAG TGG AGA TAC-3′	
HbG6PDH4-Q-R	5′-GGC TTC ATC AGG GAC ATC AC-3′	
YLS8-Q-F	5′-CCT CGT CGT CAT CCG ATT C-3′	Real-time PCR for housekeeping genes
YLS8-Q-R	5′-CAG GCA CCT CAG TGA TGT C-3′	
RH8-Q-F	5′-TCA CAG GGT TGG TAG ATC AG-3′	
RH8-Q-R	5′-CCA AGC TCT TGC TCA ATC C-3′	
RH2b-Q-F	5′-AGG TGG ATT GGC TAA CTG AG-3′	
RH2b-Q-R	5′-GAG CCC AAA CAT CAG TAG TG-3′	

### Construction of a Phylogenetic Tree

A phylogenetic tree of G6PDH was obtained by analyzing the deduced amino acid sequence from *H. brasiliensis* (AIE47266, AIE47267, AIE47268, AIE47269)*, P. trichocarpa* (EEE79649, ERP53365, EEE94856, EEF03929), *R. communis* (EEF47431, EEF32168, EEF50009), *A. thaliana* (Q9FY99, Q8I743, Q43727, Q93ZW0, Q9IK23, Q9FJI5), *Zea mays* (AFW57831, DAA45780, XP008658752, ACG39996), *O. sativa* (BAC84352, ABF96582, ABF95637, AAL79959), and *T. aestivum* (BAA97662) using the Neighbor-Joining method in the MEG 5.05 software. A bootstrap analysis was performed using 1,000 replicates.

### Subcelluar Localization

For subcellular localization analysis, the *HbG6PDH3* and *4* were sub-cloned with B*sa*I site into the pCAMBIA1302-derived pBWA(V)HS vector to produce HbG6PDH-GFP fusions (**Supplementary Figures S1** and **S2**). The specific primer pairs used were listed in **Table 1**. Leaves from rice seedlings that were cultured at 30°C in darkness after germination for 7 to 15 days were sampled to prepare protoplasts used in transient expression analysis. The experimental procedures were essential according to Yu et al. (2015). The transformed protoplasts were observed with laser scanning confocal microscope (Olympus FV1000, Janpan). For GFP, the excitation and emission wavelengths were 480 and 510 nm, respectively. Chloroplast autofluorescence was visualized in a detection channel with excitation and emission wavelengths of 556 and 650 nm, respectively.

### Hormones, Tapping, and Wounding Treatments

To determine the expression patterns of *HbG6PDHs* in response in hormone treatments, four batches of five trees were selected. Three batches were treated with each hormone in 1% carboxyl methyl cellulose (CMC) at 3, 12, and 24 h before tapping, and another batch was treated with 1% CMC as a control. The four batches were tapped at the same time, and latex was collected for RNA isolation. The hormones were jasmonic acid (JA) (0.005%), abscisic acid (ABA) (200 μmol/L), cytokinin (CTK) (200 μmol/L), salicylic acid (SA) (200 μmol/L), gibberellin (GA) (100 μmol/L), and 2, 4-dichlorophenoxyacetic acid (2, 4-D) (66 μmol/L). For the ethylene treatment, the trees were treated with 1% ethephon (2-chloroethylphosphonic acid, an ethylene generator) applied on the cut at 12, 24, and 48 h before tapping with the control group treated with 1% CMC. Latex was collected for RNA isolation and determination of enzyme activity. In the experiment where mature virgin trees were opened for tapping, fifteen trees were selected and tapped in a half spiral pattern every three days, and the latex was collected from the first eight tappings for RNA isolation and enzyme activity analysis. For the wounding treatment, four batches of 10 mature virgin trees were selected, three of which were wounded at 2, 12, and 24 h before tapping, with the fourth batch was unwounded as the control. For tapping panel dryness (TPD) experiment, four batches (five trees/each batch) were selected according to TPD severities (severity < 30%, 30% < severity < 60%, 60% < severity < 90%, with healthy trees as the control). RNA extraction for treatments involving plant hormone, tapping, wounding and TPD were carried out according to the protocol of Tang (Tang et al., 2010), while the latex collection and preparation for enzyme activity determination were as previously described (Liu et al., 2015).

### Stress Treatments

Stress treatments were carried out as previously described (Xiao et al., 2014). For the low temperature stress treatment, the tissue cultured plants were transferred to conical flasks containing Hoagland’s solution, placed in a growth chamber at 5°C under continuous white light, and incubated for 0, 3, 12, and 24 h. For the high temperature stress treatment, the manipulations were similar, except that the temperature was adjusted to 40°C, and the relative humidity was maintained at 80%. For the drought stress treatment, the tissue cultured plants were transferred into basic Hoagland’s solution containing 20% PEG6000, and incubated for a range of different times (0 h, 3 h, 6 h, 12 h, 1 day, 3 days, 4 days, and 7 days). For all the stress treatments, roots, leaves, and barks were sampled for RNA extraction from stressed plants at each time point, and samples from the unstressed plants were used as controls.

### RNA Isolation and cDNA Synthesis

Total RNA was extracted by the protocol as described previously (Tang et al., 2007, 2010). RNA samples were treated with DNase I (TaKaRa) to eliminate the trace contaminants of genomic DNA. Integrity of the RNA samples was checked by agarose gel electrophoresis, while their concentration and quality were examined by NanoDrop 2000 (Thermo, USA). Synthesis of cDNA was performed using the RevertAid^TM^ First Strand cDNA Synthesis Kit (Fermentas, Canada) following the manufacturer’s protocol.

### Real-Time PCR

Real-time PCR was performed to analyze the *HbG6PDH* expression pattern with *YLS8* (mitosis protein), *RH8* (DEAD/DEAH box helicase), and *RH2b* (DEAD box RNA helicase) as the internal control genes for normalization according to Li et al. (2011). The primers for the target and internal control genes were designed using freeware IDT DNA^1^ with default parameters, and were then synthesized by Invitrogen China (Shanghai, China) (**Table 1**). Real-time PCR was carried out using the SYBR^®^ Premix Ex Taq II (Perfect Real Time) (Takara, Dalian, China) and CFX96 Touch^TM^ Real-Time PCR Detection System (Bio-Rad, Hercules, CA, USA). The PCR reaction system and procedures were carried out as previously described (Long et al., 2015). The Bio-Rad CFX Manager Software 3.0 was used for visualizing and analyzing the data, including the quantification cycle values, PCR efficiency and correlation coefficients. The relative fold change of expression was calculated following Vandesompele (Vandesompele et al., 2002).

### Recombinant Protein Expression

To identify the enzyme activity of target proteins, recombinant vectors expressing *HbG6PDHs* were constructed to prokaryotic expression in *E. coli*. Four *HbG6PDH* ORFs were amplified using primers with different restrict enzyme sites (**Table 1**), and then inserted into the plasmid pMAL-c5E (New England BioLabs) in the same translational frame as the *malE* gene that encodes a maltose-binding protein (MBP). The resulting recombinant vectors were transformed into the *E. coli* BL21 (DE3) to generate the recombinant proteins. The transformed clones were cultivated at 37°C in liquid LB medium supplemented with 100 μg/ml ampicillin and 25 μg/ml chloramphenicol. The isopropyl β-D-1-thiogalactopyranoside (IPTG) was added at 0.4–0.6 of OD_600__nm_ to a final concentration of 1.0 mM. After shaking at 30°C for 2 h, the induced strains were broken by ultrasonic wave with 200 w for 5 min, and then centrifuged at 10,000*g* for 10 mins. The supernatant was collected and stored for protein electrophoresis detection and enzyme activity assay.

### Enzyme Activity Assays

For recombinant proteins, three independent transformed *E. coli* clones for each recombinant vector were selected to measure the enzyme activity. To analyze enzyme activity for ethylene and tapping treatments, the cytosolic serum (C-serum) was separated and purified from the latex by ultracentrifugation for 2 h at 270,000 ×*g* at 4°C G6PDH catalyze glucose-6-phosphate to 6-phosphoglucnate concomitant with the reduction of NADP^+^ to NAPDH. The G6PDH activity was measured by a spectrophotometer at 340 nm according to the increasing rate of NAPDH. One unit (U) of enzyme activity was defined as the amount of enzyme that produced 1 nmol of NAPDH in 1 min at 25°C. G6PDH activity and protein concentration were assayed using the kits purchased from SuZhou Keming Bioengineer Company (SuZhou, China), following the manufacturer’s instruction (Koide and Oda, 1959).

## Results

### Identification of *H. brasiliensis G6PDH* Genes and Functional Expression in *E. coli*

Four *G6PDH* genes were identified from the transcriptome database of rubber tree, and their full-length cDNA sequences were then obtained and submitted to GeneBank (*HbG6PDH1*, KJ599636.1; *HbG6PDH2*, KJ599637.1; *HbG6PDH3*, KJ599638.1; *HbG6PDH4*, KJ599639.1). The four *HbG6PDH* genes represent the entire rubber tree *G6PDH* family that was verified by BLAST-searching the *Hevea* draft genome sequence (Rahman et al., 2013). A homology analysis of the four full-length cDNA sequences indicated that nucleotide identity among them ranged from 40.6 to 67.2%, with the highest identity between *HbG6PDH1* and *2*, and the lowest identity between *HbG6PDH1* and *4.*

**FIGURE 1:**
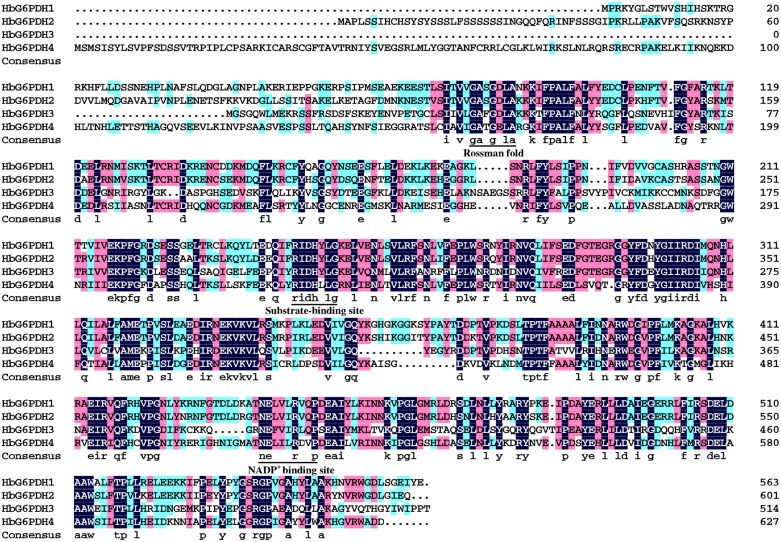
**Amino acid sequence alignment of the four *Hevea brasiliensis* glucose-6-phosphate dehydrogenase (HbG6PDH) isoforms.** Strictly conserved sequences are in white on black background; similar amino acids are in black on red and blue backgrounds. The conserved Rossman fold, substrate-binding site, NADP^+^ binding site were underlined.

The four *HbG6PDH* genes contained different length within the open reading frame (ORF) in range of 1,548 to 1,884 bp, encoding 515 to 627 amino acid residues. The predicted molecular weights of mature HbG6PDH proteins ranged from 58.9 to 70.5 kDa (**Table 2**). Reflecting what was found in the cDNA sequences, a high similarity, ranging from 45.3 to 76.1%, was shared among the four *HbG6PDH* protein sequences. Two sites, a substrate-binding site (IDHYLG) and NADP-binding site (NEFVIRLQP), were highly conserved in the protein sequences (**Figure 1**). A conserved Rossman fold (GXXGXXG/A) domain was also found in the four protein sequences (**Figure 1**). Nevertheless, high divergence was observed in some regions of the amino acid sequences.

**Table 2:** Basic information for the four *Hevea brasiliensis* glucose-6-phosphate dehydrogenase (HbG6PDHs).

Gene name	Type	Localization	a.a.	TP	MW(kDa)	GenBank
HbG6PDH1	Plastidic	Chloroplast	563	15	64.234	AIE47266
HbG6PDH2	Plastidic	Chloroplast	601	63	68.180	AIE47267
HbG6PDH3	Cytosolic	Cytosol	515	–	58.855	AIE47268
HbG6PDH4	Plastidic	Chloroplast	627	51	70.486	AIE47269

*The number of amino acids (a.a.) and molecular mass (MW) were based the proteins deduced from the full-length HbG6PDH cDNAs. Subcellular localization and transit peptide (TP) length were predicted by the online software of ProtComp 9.0 (http://linux1.softberry.com/).*

Online software, ProtComp 9.0^2^, was used to evaluate the presence of a transit peptide, which suggested the localization of HbG6PDH3 in the cytosol, and HbG6PDH1, 2 and 4 in the chloroplast (**Table 2**).

**FIGURE 2:**
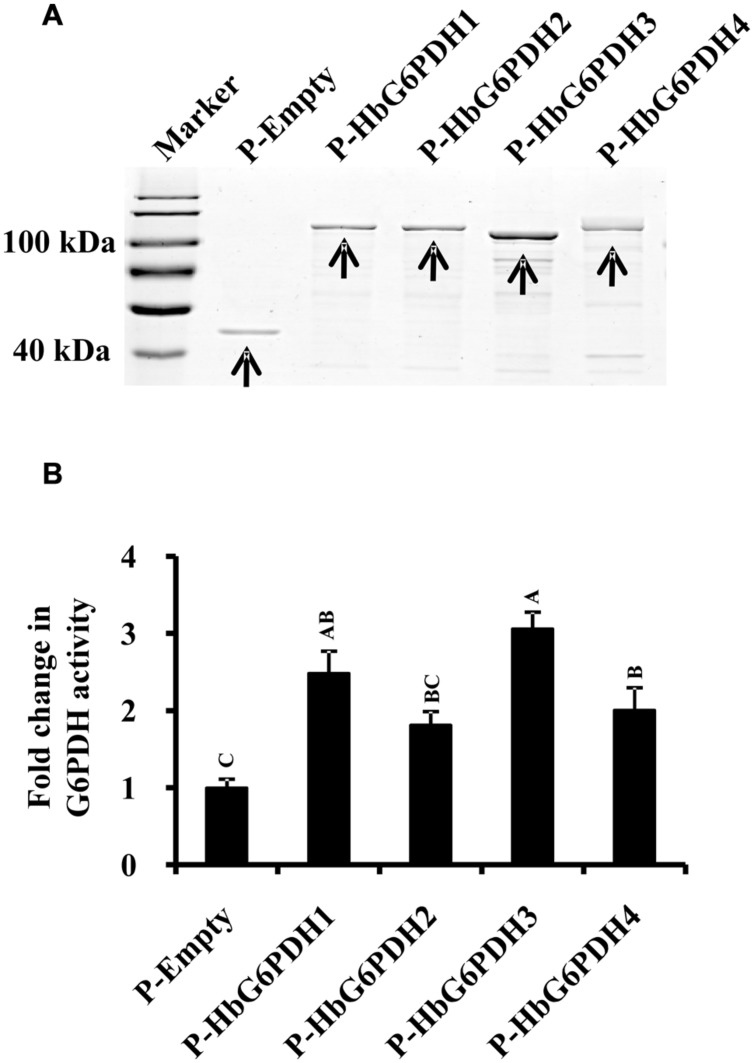
**Expression of the four *HbG6PDH* genes in *Escherichia coli*. (A)** Sodium dodecyl sulfate-polyacrylamide gel electrophoresis (SDS-PAGE) profile of protein extracts from ITPG-induced *E. coli* cells harboring the pMAL-c5E-Empty and pMAL-c5E-*HbG6PDHs* (*HbG6PDH1* to *4*). The arrowheads showed the protein bands of maltose binding protein (MBP) (P-Empty) and MBP-HbG6PDH fusions (P-HbG6PDH1 to 4). **(B)** Enzyme activity of the recombinant HbG6PDH proteins from pMAL-c5E-Empty and pMAL-c5E-HbG6PDHs (HbG6PDH1 to 4).

To determine whether these *HbG6PDHs* encode functional G6PDH enzymes, they were expressed in *E. coli* using the pMAL-c5E expression vector to produce MBP fusion that is due to express cytoplasmically. As expected, the four *HbG6PDH* genes were highly expressed in the cytosol of *E. coli*, and the recombinant proteins showed the expected molecular mass totaling the MBP protein and the respective HbG6PDH proteins (**Figure 2A**). The supernatant from the sonicated *E. coli* cells were tested for the G6PDH enzyme activity. As shown in **Figure 2B**, the extracts from *E. coli* cells expressing the four *HbG6PDH* genes showed significantly higher G6PDH activity than the control *E. coli* cells harboring an empty pMAL-c5E vector. These results indicated that all four *HbG6PDH* genes encode enzymatically active G6PDHs.

### Phylogeny and Subcellular Localization of HbG6PDHs

**FIGURE 3:**
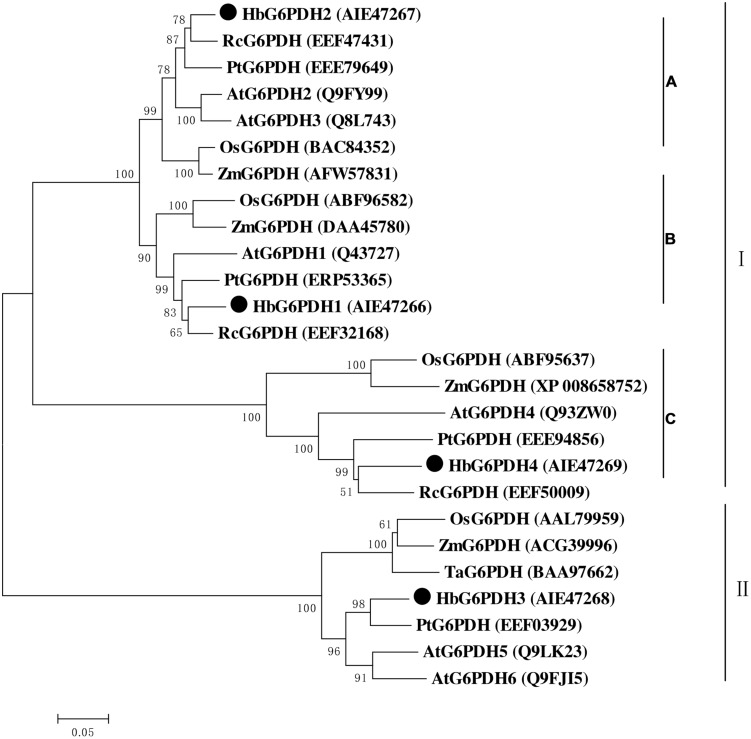
**Phylogenetic analysis of 26 plant glucose-6-phosphate dehydrogenase (G6PDH) isoforms from public databases.** The unrooted phylogenetic tree of 26 G6PDH proteins including 4 *H. brasiliensis* G6PDHs was constructed using the neighbor-joining method in the MEGA 5.05 program. Except the *H. brasiliensis*, the six other plant species are *Populus trichocarpa*, *Ricinus communis*, *Arabidopsis thaliana*, *Zea mays*, *Oryza sativa*, and *Triticum aestivum*. The group I were divided into three clusters **(A–C)**.

**FIGURE 4:**
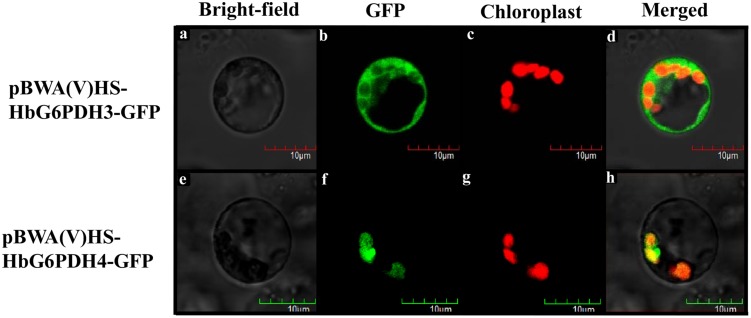
**Localization of pCAMBIA1300-HbG6PDH3-GFP and pCAMBIA1300-HbG6PDH4-GFP in rice protoplasts.** Scale bar = 10 μm. **(a,e)**, bright field image; **(b,f)**, transient expression of GFP; **(c,g)**, chloroplast autofluorescence; **(d,h)**, merged GFP and chloroplast image.

Multiple sequence alignment was performed among four HbG6PDHs and other twenty-two G6PDH proteins of six plants, *viz. P. trichocarpa*, *R. communis*, *A. thaliana, Z. mays*, *O. stativa*, and *T. aestivum.* The results showed 35.4–92.4% homology in amino acid sequence among the G6PDH members, and the substrate-binding site and NADP-binding site were both highly conserved in these G6PDHs plants. However, the transit peptides associated with the different proteins were diverse, leading to varying predicted locations of the G6PDH family members. A phylogenic tree was constructed on the basis of sequence similarity of the mature proteins, and classified them into two groups (I and II) (**Figure 3**). Group I corresponded to plastidic G6PDH isoforms including four *Arabidopsis* plastidic isoforms (AtG6PDH1 to 4) (Wakao and Benning, 2005; Née et al., 2009). Group I was further divided into three clusters (A, B, and C), into which the three HbG6PDHs (HbG6PDH1, 2 and 4) were equally scattered. It is worth noting that in each cluster, the HbG6PDH isoform was closely related to its homologue from *R. communis* under the same spurge (Euphorbiaceae) family with rubber tree. Group II corresponded to the cytosolic isoforms, including HbG6PDH3 and the two *Arabidopsis* cytosolic isoforms (AtG6PDH5 and 6) which have been experimentally verified to locate in cytosol (Wakao and Benning, 2005; Wakao et al., 2008). The phylogenic analysis is consistent with the previous online prediction for the HbG6PDHs. To further verify the subcellular locations of the HbG6PDHs, two latex-abundant isoforms, *HbG6PDH3* and *4* were translationally fused to GFP, and transiently expressed in rice protoplasts. As shown in **Figure 4**, HbG6PDH3 and 4 proteins were unequivocally located in cytosol and chloroplast, respectively.

### Tissue Expression Analysis of *HbG6PDHs*

**FIGURE 5:**
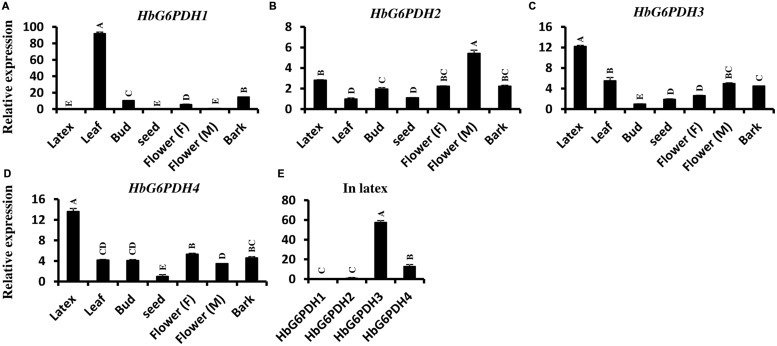
**Expression of four *HbG6PDH* isoforms in different tissues. (A)** *HbG6PDH1*; **(B)** *HbG6PDH2*; **(C)** *HbG6PDH3*; **(D)** *HbG6PDH4*; **(E)** Relative expression of four *HbG6PDH* isoforms in the latex. Flower (M) = male flower; Flower (F) = female flower. Different capital letters indicate a significant difference at 0.01 level.

The transcript levels of *HbG6PDH* genes were investigated in seven *Hevea* tissues (**Figures 5A–D**). Among the four genes, *HbG6PDH3 and HbG6PDH4* showed a basically similar pattern of expression (**Figures 5C,D**). They were much higher (5- to 10-folds) expressed in latex than in other tissues. *HbG6PDH1* presented somewhat a leaf-specific expression pattern, with the highest expression level in leaves, but with low or very low levels in the other tissues (**Figure 5A**). *HbG6PDH2* showed a relatively constitutive expression; having much less variation among different tissues compared with other *HbG6PDH* genes (**Figure 5B**). Further, the relative expression abundance of the four *HbG6PDH* genes in latex was also analyzed. The results showed that *HbG6PDH3* was the major isoform in latex, its expression being 50 fold more than that in *HbG6PDH1* and *2* (**Figure 5E**). Although *HbG6PDH4* expression in latex was only about 20% of that in *HbG6PDH3*, it was still much higher than the expression of the other two genes. Hereafter, the gene expression studies in latex, cytoplasm of rubber-producing laticifers will be focused on *HbG6PDH3* and *4*.

### Expression of *HbG6PDH3* and *4* in Latex in Response to Tapping, Ethylene, Wounding, and TPD

**FIGURE 6:**
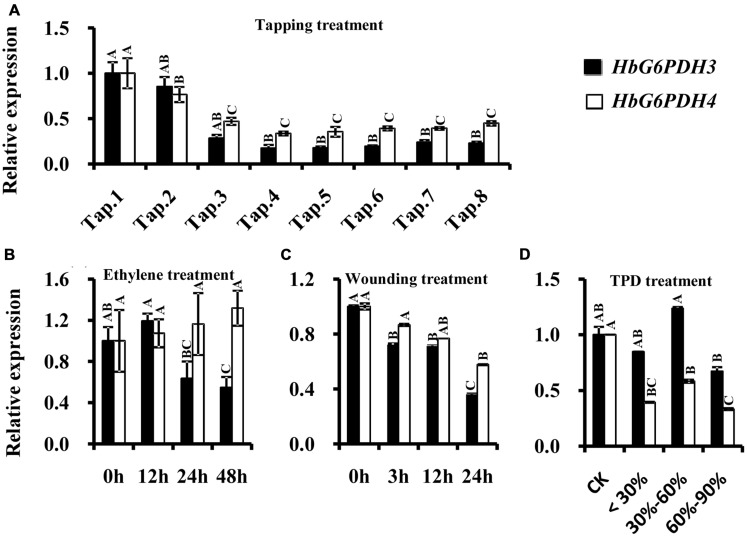
**Responses of *HbG6PDH3 and HbG6PDH4* to tapping (A), ethylene (B), wounding (C), and different severities of tapping panel dryness (TPD), (D).** Different capital letters indicate a significant difference at 0.01 level for the same gene.

In the latex of rubber trees, the expression profile of *HbG6PDH3* and *4* were investigated after tapping, wounding, and ethylene application. Comparisons were also made between latex from trees suffering from TPD and latex from healthy trees. In the tapping experiment, *HbG6PDH* expression levels sharply declined in the first four tappings, and remained low thereafter. Compared with *HbG6PDH4*, the expression of *HbG6PDH3* was more obviously affected by tapping, showing a 6-fold change in transcript levels in *HbG6PDH3* as compared with a 3-fold with *HbG6PDH4* after a series of tappings (**Figure 6A**). Following ethylene simulation, *HbG6PDH3* and *HbG6PDH4* showed different expression patterns, and the expression changes were much more obvious in *HbG6PDH3* (**Figure 6B**). The expression of *HbG6PDH3* was increased at 12 h treatment, and then declined abruptly thereafter. In comparison, there was little variation in the expression of *HbG6PDH4* across treatments. After the wounding treatment, *HbG6PDH3* and *HbG6PDH4* expressions exhibited a similar profile of down-regulation, except that the extent of *HbG6PDH3* down-regulation was more marked (5.8-folds) (**Figure 6C**). TPD is a complex physiological disorder that affects latex regeneration (Venkatachalam et al., 2009). In rubber trees with different degrees of TPD, the transcripts of *HbG6PDH4* were significantly higher in healthy trees than in TPD ones (**Figure 6D**). However, there was no clear correlation between the expressional levels of the *HbG6PDH3* and TPD severity.

### G6PDH Enzyme Activity in Latex Following Ethylene and Tapping Treatments

**FIGURE 7:**
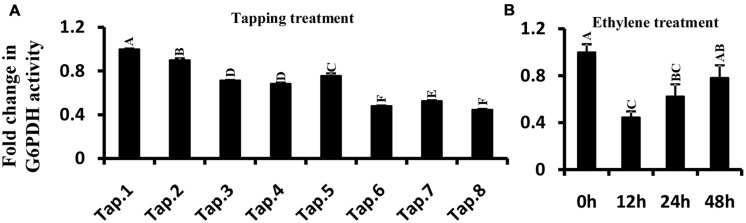
**Effect of tapping (A) and ethylene treatment (B) on *Hevea brasiliensis* G6PDH enzyme activity in latex.** Different capital letters indicate a significant difference at 0.01 level.

Glucose-6-phosphate dehydrogenase enzyme activity was measured in latex when trees were first tapped, and also following treatment with ethylene. In tapping experiment carried out on previously untapped trees, the enzyme activity gradually decreased with tapping, reaching about half of the enzyme activity recorded at the first tapping (**Figure 7A**). The trend in G6PDH enzyme activity with successive tappings was consistent with that of *HbG6PDH3* and *4* gene expression (**Figures 6A and 7A**). With ethylene treatment, G6PDH enzyme activity decreased markedly after 12 h, but recovered gradually over 24 to 48 h from the time of ethylene application (**Figure 7B**). The change of enzyme activity did not show any obvious relationship with gene expression of either *HbG6PDH3* or *4* (**Figures 6B and 7B**), suggesting a posttranscriptional and/or posttranslational regulation of ethylene treatment on the G6PDH activity.

### Expression of *HbG6PDH3* and *4* in Latex Following Treatments with Plant Hormones

**FIGURE 8:**
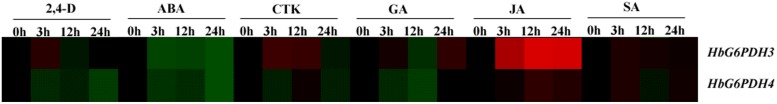
**Responses of *HbG6PDH3 and HbG6PDH4* to six plant hormones, *viz.* 2, 4-dichlorophenoxyacetic acid (2, 4-D), abscisic acid (ABA), cytokinin (CTK), gibberellic (GA), jasmonic acid (JA), and salicylic acid (SA).** The expression data are shown as fold change (log_2_) as calculated by the ratio between the control (0 h) and treatments (3, 12, and 24 h) for each gene. Different colors represent different changes in the *HbG6PDH* gene expression levels: black represents no change, green represents down-regulation and red represents up-regulation. The brightness indicates the degree of expression change.

The expression patterns of *HbG6PDH3* and *4* were investigated in latex after treatment with six hormones, *viz.* 2, 4-dichlorophenoxyacetic acid (2, 4-D), ABA, CTK, GA, JA, and SA (**Figure 8**). The two *G6PDH* genes presented generally similar expression patterns after ABA and JA treatments: down-regulation by ABA and up-regulation by JA, respectively. In the case of GA, the expression of *HbG6PDH4* showed an initial down-regulation followed by a reversal, whereas *HbG6PDH3* expression fluctuated during 24h treatment. After SA treatment, the expression of *HbG6PDH3* increased for the first 12 h, and decreased thereafter; *HbG6PDH4* expression showed an irregular response. In comparison, JA treatment showed a much higher stimulatory effect on the expression of *HbG6PDH3* than on *HbG6PDH4*. In addition, *HbG6PDH3* and *4* showed different expression patterns in response to the treatment of 2, 4-D and CTK. After 2, 4-D treatment, the expression of *HbG6PDH3* showed a transient up-regulation at 3 h, whereas the expression of *HbG6PDH4* decreased with the treatment, reaching the lowest level after 24 h. The expression of *HbG6PDH3* displayed an increase for 12 h following CKT treatment, and then an obvious decrease after 24 h. In contrast, the expression of *HbG6PDH4* showed a down-regulation after CTK treatment. The distinct response patterns of the *HbG6PDH* genes to hormone treatments suggest their differing roles in hormone-regulated metabolism.

### Expression of *HbG6PDHs* in *Hevea* Seedlings Responding to Abiotic Stresses

To understand the responses of *HbG6PDHs* to temperature and drought stresses, the expression profiles of four genes were examined in root, bark, and leaf of *Hevea* seedlings after stress treatment.

**FIGURE 9:**
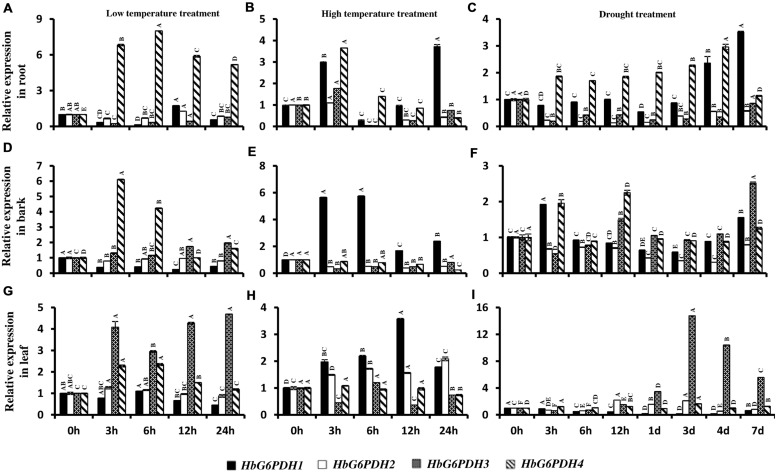
**Expression of *HbG6PDH* isoforms in the root, bark, and leaves in response to various stress treatments, *viz.* low temperature (A,D,G), high temperature (B,E,H) and drought (C,F,I).** Readings are expressed as values relative to the unstressed control. Different capital letters indicate a significant difference at 0.01 level among different samples for the same gene.

In root, the four *HbG6PDH* genes showed obvious responses to stresses of low temperature, high temperature, and drought. During low temperature treatment, *HbG6PDH1, 2 and 3* showed similar patterns of an initial falling and then gradual rising expression (**Figure 9A**). However, *HbG6PDH4* expression was strongly induced quickly after low temperature treatment, reaching the highest level at 6 h, and decreasing slightly thereafter (**Figure 9A**). After high temperature treatment, *HbG6PDH1* to *3* showed similar expression dynamics: initial up-regulation at 3 h, then abrupt down-regulation at 6 h, and finally a recovery, although the extent of their expression change differed (**Figure 9B**). *HbG6PDH4* expression showed a different response to the high temperature treatment, which was up-regulated at 3 h, and then decreased gradually. After drought treatment, *HbG6PDH1* and *HbG6PDH4* expression gradually rose with the duration of treatment (**Figure 9C**). The other two genes, *HbG6PDH2* and *HbG6PDH3*, were up-regulated first and then down-regulated by drought treatment (**Figure 9C**).

In bark, the four *HbG6PDH* genes also presented obvious responses to temperature and drought treatments. After low temperature treatment, *HbG6PDH1* expression declined steadily, and *HbG6PDH4* expression first increased, then decreased (**Figure 9D**). In comparison, the other two genes (*HbG6PDH2* and 3) showed little response to low temperature treatment. After high temperature treatment, the expressions of *HbG6PDH2 to 4* were down-regulated, whereas the expression of *HbG6PDH1* was bolstered and peaked at 6 h before sharply receding thereafter (**Figure 9E**). After drought treatment, the expressions of the four *HbG6PDH* genes fluctuated through the duration of 7 day treatment (**Figure 9F**).

In leaves, the four *HbG6PDH* genes showed distinct expression patterns in response to three types of stresses. After low temperature treatment (**Figure 9G**), *HbG6PDH3* and *4* expressions were up-regulated, although the extent of up-regulation was much higher for the former. Over the duration of treatment, the expression of *HbG6PDH1* was decreased, whereas *HbG6PDH2* was little affected. During high temperature treatment, *HbG6PDH1* expression was up-regulated with peaking at 12 h, but the other three genes had no response or irregular fluctuation in expression (**Figure 9H**). *HbG6PDH3* was obviously induced by drought treatment; its expression remained at a low level during the early stage of treatment but sharply increased from 12 h and peaked at 3 days (**Figure 9I**). *HbG6PDH1* expression exhibited a progressive down-regulation, reaching its lowest level at 4 days. The expression trend of *HbG6PDH2* was inconsistent, falling, and rising over time. Compared with the other three genes, the expression of *HbG6PDH4* was little affected by drought treatment (**Figure 9I**).

## Discussion

In plants, the PPP is an important carbohydrate metabolic pathway that generates NADPH and pentoses (5-carbon sugars), in parallel to the glycolytic pathway (Dennis et al., 1997; Kruger and von Schaewen, 2003). As one of key enzymes in the PPP, G6PDH controls carbon flow and NADPH production, playing an important role in plant growth and development. The pathway also plays a role in the response of plants to biotic and abiotic stresses.

### Rubber Tree has Four *G6PDH* Genes

Consistent with other plants, rubber tree has a small G6PDH gene family. In this study, the four rubber tree *G6PDH* genes (*HbG6PDH1* to *4*) were first identified basing on a comprehensive *H. brasiliensis* transcriptome, and then verified as the entire G6PDH gene family after blasting against the recently released rubber draft genome sequence (Rahman et al., 2013). The deduced protein sequences have two highly conserved sites for substrate-binding (IDHYLG) and NADP-binding (NEFVIRLQP) (**Figure 1**), suggesting the corresponding genes encode active G6PDH enzymes. This kind of prediction was later demonstrated by investigating the enzymatic activities of the recombinant HbG6PDH proteins expressed in *E. coli* (**Figure 2**). Two groups of G6PDH isoforms, plastidic, and cytosolic G6PDH, are found in rubber tree of which HbG6PDH3 is cytosolic, and the other three HbG6PDHs (HbG6PDH1, 2 and 4) are plastidic. The larger number of plastidic isoforms indicates that most PPP steps take place in plastids rather than the cytosol in rubber tree, a phenomenon commonly observed in the plant kingdom (Kruger and von Schaewen, 2003). The subcellular localizations of these HbG6PDHs were initially predicted by an online software platform and consolidated by a phylogenetic analysis (**Table 2**; **Figure 3**), and subsequently demonstrated by transient expression of HbG6PDH3 and 4 in rice protoplasts using GFP fusions (**Table 2**; **Figures 3** and **4**). The divergence of G6PDH into cytosolic and plastidic types may have occurred before species divergence in plants, and plays a role in adaptation of plants to various habitats.

Different isoforms of G6PDH function in respective functional areas in plants. For example, the cytosolic G6PDHs are widespread in all tissues, especially high expression in sink tissues, whereas plastidic ones are highly expressed in green tissues and less so in sink tissues (von Schaewen et al., 1995). In our study, the plastidic *HbG6PDH1* had a tissue-specific expression pattern with the highest expression level in leaves, but was low or very low expressed in a variety of sink tissues (**Figure 5A**), consistent with its *Arabidopsis* and Nicotiana orthologues (Knight et al., 2001; Wakao and Benning, 2005). However, the other two plastidic isoforms, *HbG6PDH2* and *4*, and the cytosolic *HbG6PDH3* were abundantly expressed in different Hevea tissues. These results suggest that *HbG6PDH1* functions mainly in green tissue (leaves), whereas the other three *HbG6PDH* genes function in diverse source and sink tissues (**Figures 5B–D**). Besides, the expression of *HbG6PDH3* and *4* in latex had varying responses to different plant hormones, suggesting their distinct roles in various signal transduction pathways (**Figure 8**). Generally, *HbG6PDH3* is more responsive to hormones than *HbG6PDH4*, and the expressions of both genes display little change in fold to most hormones. Of the six hormones and two *HbG6PDH* genes examined, a >2-fold expressional change was observed only for JA on *HbG6PDH3*, suggesting the involvement of *HbG6PDH3* in JA responses in laticifers.

### HbG6PDH3 and 4 are Implicated in PPP During Latex Regeneration

Nicotinamide adenine dinucleotide phosphate is mainly derived from photosynthesis and widely used in several anabolic reactions such as carbon fixation, fatty acid synthesis, and nitrogen assimilation (Dennis et al., 1997). In non-photosynthetic tissues or under non-photosynthetic conditions, the PPP is regarded as major sources of NADPH used for basic metabolism during plant growth and development (Thom et al., 1998). Beyond that, the PPP also provides abundant intermediary metabolites for other anabolic metabolisms, including the synthesis of aromatic amino acids, cell wall, and pigments (von Schaewen et al., 1995; Hauschild and von Schaewen, 2003) and is involved in many stress responses (Yang et al., 2014). As a rate-limiting enzyme in the PPP, G6PDH studies are always mainly focused on carbohydrate metabolism. Previous research has shown that G6PDH plays an important role in seed development, where the enhancement of its enzyme activity lead to the breaking of seed dormancy and the improvement of seed germination (Pu et al., 1994; Han et al., 1998). Also, the expression and activity of G6PDH is closely related with young spike development in rice (Huang et al., 2003). In peach, the G6PDH provides precursors for metabolic processes responsible for the red coloration of fruit (Kong et al., 2007).

In rubber tree, synthesis of natural rubber in laticifers uses sucrose as the main raw material (Tupy, 1973). The uptake of sucrose into laticifers relies on sucrose transporters, of which HbSUT3 is the major responsible member (Tang et al., 2010), and its cleavage into fructose and glucose in latex is conducted by a cytoplasmic alkaline/neutral invertase (HbNIN2) (Liu et al., 2015). The resultant glucose is metabolized by two parallel pathways, the PPP and the glycolytic pathway for providing metabolites (NADPH and 5-carbon sugars) and energy exploited in latex regeneration (Tupy, 1973). Of the four *HbG6PDH* genes, *HbG6PDH3* and *HbG6PDH4* are the major isoforms expressed in latex, and both have much higher expression levels in latex than the other tissues (**Figures 5C,D**), suggesting their major roles in the PPP in latex. In comparison, expression of the cytosolic *HbG6PDH3* is more than five-fold that of the plastidic *HbG6PDH4* (**Figure 5E**), indicating the relative importance of cytosolic PPP in latex metabolism. The HbG6PDH4 protein was predicted to locate on Frey-Wyssling particles, the sole class of plastidic type organelles suspended in latex. Both G6PDH enzyme activity and expression of *HbG6PDH3* and *HbG6PDH4* displayed striking responses to tapping in latex of virgin rubber trees (**Figures 6A and 7A**), and the pattern of these responses are negatively correlated with the tapping-stimulated rubber production (Tang et al., 2010), which suggest the involvement of PPP in latex regeneration.

Ethylene is a commonly used latex yield stimulant in rubber production. Our results showed that ethylene depressed both *HbG6PDH3* expression and G6PDH enzyme activity (**Figures 6B and 7B**). However, compared with the effect of tapping on virgin rubber trees, Ethylene treatment posed a much lesser effect on *G6PDH* gene expression and enzyme activity, which is consistent with its lesser effect on the latex output. Compared with *HbG6PDH4*, the latex-predominant isoform *HbG6PDH3* showed a much more marked response to either tapping or the ethylene treatment (**Figures 6A,B**). Previous studies have shown that cytosolic G6PDH is the major isoform in higher plants, accounting for 80–95% of the whole cellular activity (Cardi et al., 2013). Based on the above results, we conclude that the cytosolic HbG6PDH3 is the major isoform functioning in *Hevea* latex regeneration. Although the exact mechanisms remain to be investigated, we could speculate the contribution of HbG6PDH3 to latex regeneration at least from two aspects: (1) the synthesis of NADPH and 5-carbon sugars; (2) the regulation of reactive oxygen species (ROS) metabolism. NADPH and 5-carbon sugars are essential for latex regeneration that includes various anabolic metabolisms, e.g., rubber biosynthesis and nucleic acid synthesis. Rubber production is essentially a defense response, and moderate levels of ROS production will help induce defense responses and thus contribute to latex production (D’Auzac et al., 1997; Duan et al., 2010). The tapping- and ethylene-depressed *HbG6PDH3* expression and G6PDH enzyme activity (**Figures 6** and **7**) will contribute to increased ROS production (Leopold et al., 2003; Mendez et al., 2011), thus stimulating latex production. The potential roles of *HbG6PDH3* in latex regeneration were further supported by the marked simulating effect of JA on *HbG6PDH3* expression during 24 h treatment (**Figure 8**). JA is the sole phytohormone known to induce laticifer differentiation, which constitutes one of the key factors restricting sustainable rubber production (Hao and Wu, 2000). The up-regulation of *HbG6PDH3* by JA suggested its additional contribution to latex regeneration through the participation in JA-stimulated laticifer differentiation. Also, it is worthwhile to investigate the functions of HbG6PDH4 in the anabolic metabolisms occurring in the Frey-Wyssling particles that contain carotenoid pigments. The results will help address not only the biological roles of Frey-Wyssling particles, but their functions in the regulation of latex regeneration.

### *HbG6PDHs* are Responsive to Various Stresses

In plants, G6PDH is induced by both biotic and abiotic stresses. In this regard, the enzyme plays a key role in maintaining redox balance and protecting cells from the harm caused by oxidative stress. Common environment stresses cause excessive ROS generation and accumulation, which are harmful to cellular structure and function in plant. NADPH not only acts to maintain the output of reduced glutathione (GSH), but is also involved in activity of plasma membrane (PM) NADPH oxidase which would result in hydrogen peroxide (H_2_O_2_) accumulation (Leopold et al., 2003; Mendez et al., 2011). Any decrease in G6PDH activity upsets internal redox balance, leading to disorders of signaling pathways and cell cycles in plant (Manco et al., 2011). G6DPH is a key enzyme in rice, responding to salt stress to maintain cell redox balance (Zhang et al., 2013). In soybean roots, G6PDH plays a central role in the process of H_2_O_2_ metabolism to maintain GSH and ascorbate (Asc) levels under drought stress (Liu et al., 2013). In sugarcane, assay of both activity and transcription level of ScG6PDH indicate that cytosolic ScG6PDH plays a positive role in response to various stresses, including salt, drought, heavy metal (CdCl_2_), and low temperature treatments (Yang et al., 2014).

In rubber production, every tapping is a wounding treatment for rubber tree, which causes oxidative stress that influences physiological metabolism in laticifers. The overtapping and/or overstimulation of rubber trees is thought to result in a complex physiological disorder known as TPD where latex coagulates in the laticifers and the tree stops yielding partially or completely (Sookmark et al., 2002; Venkatachalam et al., 2009). In this study, *HbG6PDH 4* were implicated in defending TPD, with high expression levels in the healthy tree (**Figure 6D**). Both *HbG6PDH3* and *4* showed an obvious decreasing expression response to wounding treatment, which might depress the metabolic rate of the PPP (**Figure 6C**). In latex, peroxides may build up when the PPP activity declines in the course of latex flow when tree is tapped. It is speculated that HbG6PDHs reduce peroxide in latex to maintain redox balance and relieve oxidative stress during every tapping. In present study, *HbG6PDHs* were induced under different environmental stresses, including drought and temperature stresses (**Figure 9**). Responses of *HbG6PDHs* to drought were more intense than to temperature stress, especially for *HbG6PDH3* in leaves and *HbG6PDH1* in root. Under low temperature stress, *HbG6PDH4* had similar up-regulated expression pattern in root, bark and leaf, suggesting its roles in protecting against the effects of low temperature. Different *HbG6PDHs* responded differently to stresses, suggesting the regulation of stress responses in rubber tree by a variety of factors involving many signal pathways.

## Author Contributions

XL designed the development of this study, carried out the experiments, and wrote the manuscript. BH participated and analyzed data in the experiment. YF carried out sequence alignment analysis. CT planned the study, formulated the question, and participated in the design of the experiments.

## Conflict of Interest Statement

The authors declare that the research was conducted in the absence of any commercial or financial relationships that could be construed as a potential conflict of interest.

## Footnotes

1 http://sg.idtdna.com/calc/analyzer

2 http://linux1.softberry.com/

## Supplementary material

FIGURE S1 **The vector of HbG6PDH3 for subcelluar localization**.

FIGURE S2 **The vector of HbG6PDH4 for subcelluar localization**.
