# Supplementary figures and images for: Identification and Characterization of the Glucose-6-Phosphate Dehydrogenase Gene Family in the Para Rubber Tree, Hevea brasiliensis
Source: Front Plant Sci. 2016 Feb 25;7:215. doi: 10.3389/fpls.2016.00215 (PMC4766392; doi:10.3389/fpls.2016.00215)

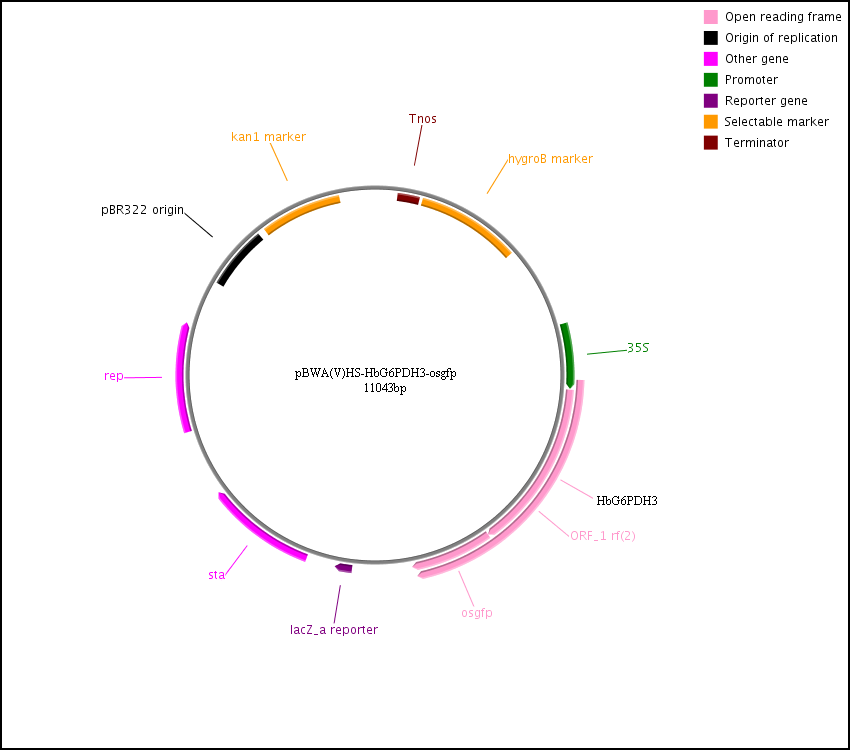

Supplement: FIGURE S1 — The vector of HbG6PDH3 for subcelluar localization. [file Image_1.TIF]

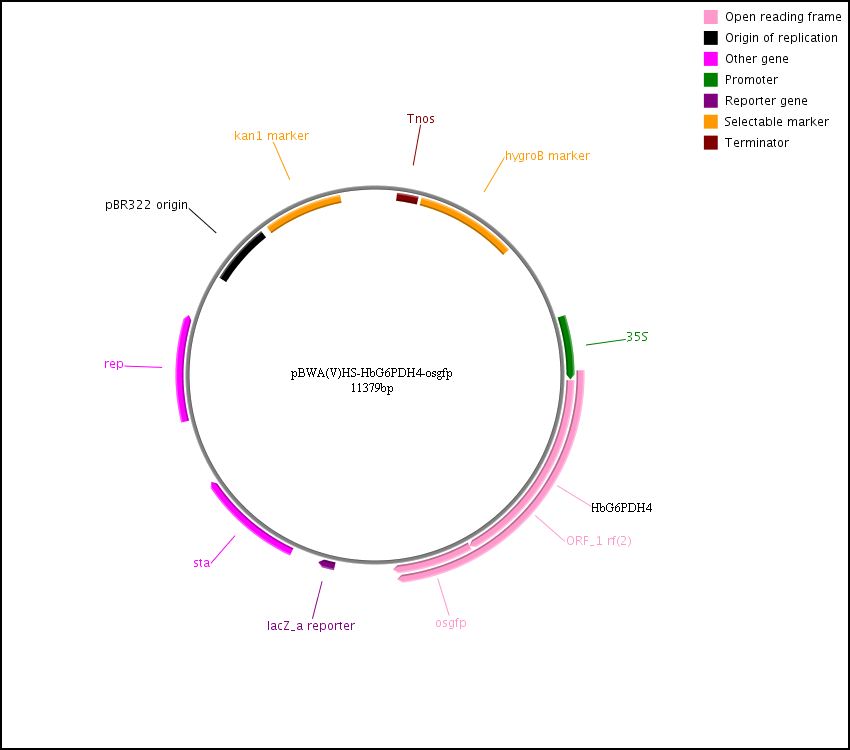

Supplement: FIGURE S2 — The vector of HbG6PDH4 for subcelluar localization. [file Image_2.TIF]
